# The association of infant feeding patterns with food allergy symptoms and food allergy in early childhood

**DOI:** 10.1186/s13006-019-0241-x

**Published:** 2019-10-24

**Authors:** Joacy G. Mathias, Hongmei Zhang, Nelis Soto-Ramirez, Wilfried Karmaus

**Affiliations:** 1 Division of Epidemiology, Biostatistics, and Environmental Health, School of Public Health, The University of Memphis, Memphis, TN USA; 2 College of Social Work, University of South Carolina, Columbia, SC USA

**Keywords:** Breastfeeding, Food allergy, Feeding modes, Formula feeding, Mixed feeding, Infant feeding, Food allergy symptoms

## Abstract

**Background:**

The role of infant feeding for food allergy in children is unclear and studies have not addressed simultaneous exposures to different foods. The goal of this study was to analyze existing data on feeding practices that represent realistic exposure and assess the risk of food allergy symptoms and food allergy in children.

**Methods:**

The Infant Feeding Practices Study II conducted by the CDC and US-FDA enrolled pregnant women and collected infant feeding information using nine repeated surveys. Participants were re-contacted after 6 years. Food allergy data were collected at 4, 9, 12, and 72 months. In total, 1387 participants had complete infant feeding pattern data for 6 months and information on food allergy symptoms and doctors’ diagnosed food allergy. Feeding patterns constituted six groups: 3-months of feeding at breast followed by mixed feeding, 3-months of breast milk and bottled milk followed by mixed feeding, 1-month of feeding at breast followed by mixed feeding, 6-months of mixed feeding i.e., concurrent feeding of breast milk, bottled milk and formula, 2–3 months of formula followed by formula and solid food, and formula and solid food since the first month. To estimate risks of food allergy, we used linear mixed models, controlling for potential confounders.

**Results:**

Of the 328 children with food allergy symptoms in infancy and at 6 years, 52 had persistent symptoms from infancy. Children exposed to mixed feeding had a higher risk of food allergy symptoms (Risk Ratio [RR] 1.54; 95% Confidence Interval [CI] 1.04, 2.29) compared to 3-months of feeding at breast adjusted for confounding. No statistically significant risk of infant feeding patterns was found for doctors’ diagnosed food allergy. Paternal allergy posed a higher risk for food allergy symptoms (RR 1.36; 95% CI 1.01, 1.83). Prenatal maternal smoking increased the risk for doctors’ diagnosed food allergy (RR 2.97; 95% CI 1.53, 5.79).

**Conclusions:**

Analysis of this prospective birth cohort suggest that introduction of multiple feeding source may lead to food allergy symptoms. Future efforts are needed to determine acceptable approaches to improve the ascertainment of food allergy in children and the role of infant feeding.

## Background

Food allergy has been on rise in developed countries [1, 2]. Between 1997 and 2007, the prevalence of food allergy has increased from 3.9% to approximately 5.0% in the United States (U.S.) [3]. Children younger than 6 years of age, especially, have a higher prevalence of food allergy when compared to children aged 6 to 17 years (4.7% vs 3.7%) [4]. In 2016, a cross sectional study of 333,200 children up to 5 years of age reported provider-diagnosed food allergy prevalence to be 6.7% [5].

In a sensitized individual, ingestion of food protein allergens could give rise to pathological immune reactions, causing clinical symptoms that range in their severity from mild itching to severe anaphylaxis which could be fatal. Children with food allergy are also at an increased risk for asthma and wheezing [4]. Furthermore, food allergy management is expensive. The U.S. health care system spends approximately $24.8 billion, annually, on food allergy [3]. Unfortunately, there is no cure for food allergy yet, nor have the mechanisms for food allergy been fully understood [3, 6]. Therefore, regarding prevalence, comorbidities, and costs, effective methods of prevention and treatment are of substantial value.

Although, some studies suggest a protective effect of breastfeeding [5–14], in a recent report by the American Academy of Pediatrics, it was concluded that the association between duration of breastfeeding and food allergy incidence in early childhood is unclear [15]. Therefore, the relationship between various infant feeding patterns (IFP), and the risk of food allergy has been controversial [14–21].

The controversial findings may be explained by divergent information on feeding indicators that are used when associating IFP to food allergy [16]. For instance, as per WHO, “exclusive breastfeeding” includes breastmilk from his/her mother or pumped breastmilk with no other liquids. The term “predominant breastfeeding” is similar to exclusive breastfeeding with an addition of water and water-based drinks. “Bottle feeding” constitutes feeding liquid or semi-solid food from a bottle with a nipple/teat, also including breastmilk in a bottle [22].

These definitions raise concerns posing a challenge to risk assessments [22–24]. First, definitions overlap with each other. For example, the definition of bottle feeding overlays with exclusive breastfeeding and predominant breastfeeding as both definitions comprise expressed milk. Second, feeding at the breast is not differentiated from pumping and feeding (expressed milk). Third, when estimating risks, infant feeding studies often focus on the duration of (exclusive) breastfeeding, but did not take the matrix of various patterns of feeding into account [13, 25–28]. While it is difficult to express a changing matrix of exposures as duration, it represents a realistic scenario of feeding an infant may be exposed to.

Therefore, the goal of the current study was to address the challenge of muddled feeding indicators by regrouping feeding practices to possibly represent realistic feeding patterns a mother may employ and assess the risk of food allergy in children. Empirical assessment of the infant feeding modes instead of the traditional duration of breastfeeding showed increased risks for asthma and eczema in early childhood [29, 30]. Specifically, mixed modes of feeding, i.e. a concurrent application of at breast feeding, bottled milk and solid food, showed an increased risk for eczema in early childhood when compared to infants who were fed at the breast [29]. Given that food allergy, eczema, asthma and rhinitis belong to atopic diseases of childhood [31], we hypothesized that mixed modes of feeding may also result in an increased risk for food allergy. To this end, we analyzed data of the Infant Feeding Practices Study II (IFPSII) and its 6 year follow up (Y6FU). This data provided repeated measurements over time of IFP in the first 6 months, multiple measurements of food allergy symptoms at different time points, and doctor diagnoses. Additionally, we also investigated whether the same children were repeatedly affected, and whether the children were taken to their medical provider to deal with food allergy symptoms.

## Methods

### Study data

Centers for Disease Control and Prevention (CDC) along with the US Food and Drug Administration (FDA) conducted a longitudinal survey called IFPS II, from May 2005 to June 2007. The sampling frame for the IFPS II constituted a consumer opinion panel of > 500,000 households in the US. The survey sample consisted of about 4000 healthy, pregnant women who were nationally distributed and in their third trimester. In the first year of the infant’s life, feeding practice information was available for about 2000 of the mother-infant dyads. Mother-infant pairs were excluded from the study if (i) mother or the infant had a medical condition at birth that would impede feeding, (ii) the infants’ gestational age was < 35 weeks, (iii) the birth weight of the infant was < 5 lb., (iv) infant was not a singleton, and (v) infant was treated in intensive care for more than 3 days. These exclusions were based on a postnatal birth screener. Since the participants were not subjected to an intervention or experiment, return of the completed initial questionnaire which contained information about informed consent procedure was considered an informed consent.

Information on postnatal birth screener were gathered through short telephone interviews. Once qualified, mothers were mailed self-reporting questionnaires. Feeding information was collected nine times in the first year of life, every month from months two to seven and then every 7 weeks until month 12. Questionnaires were validated through a series of cognitive and practical assessments that included approval from expert panel members from FDA and four pilot tests in the aforementioned sampling frame. In 2012, when the offspring were 6 years old, CDC and FDA conducted a follow-up study of the children from IFPSII called Y6FU. Demographics, feeding, health status, socio-economic related information were included in IFPSII questionnaires; health related information of the children who participated in IFPSII were the focus of Y6FU questionnaires. Information on food allergy was collected four times at four, nine, 12 months in IFPSII and in Y6FU questionnaires. The response proportion ranged from 63 to 83% for each of the questionnaire. Details about the study population can be found elsewhere [32–34].

### Exposure variables

**Fig. 1:**
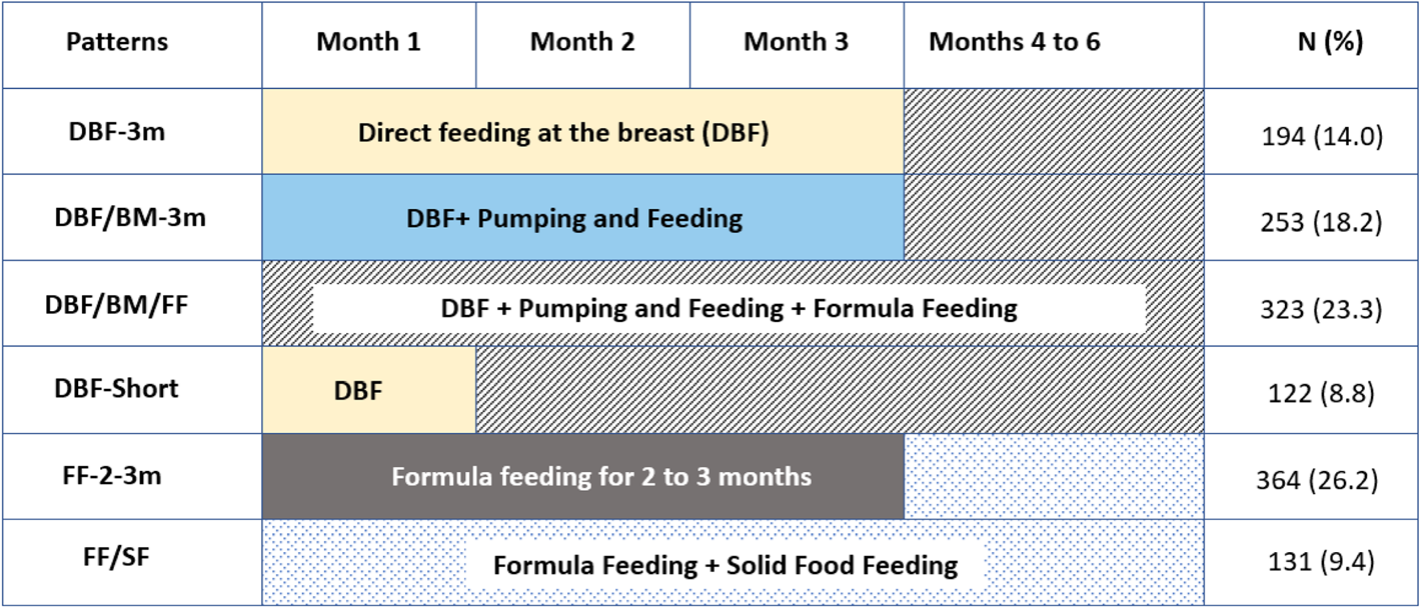
Patterns of infant feeding in the first 6 months of life. DBF-3 m - Direct feeding at the breast i.e., feeding directly at the breast for at least 3 months, not including pumping methods or any other additional food or liquid, followed by mixed feeding - this group constituted our reference group; DBF/BM - Direct feeding at the breast as well as pumping and feeding includes direct feeding at the breast and feeding of stored breast milk (BM) for the first 3 months, followed by mixed feeding; DBF/BM/FF - Concurrent application of direct feeding at the breast, pumping and feeding and formula feeding in the first 6 months; DBFShort - Direct feeding at the breast for a month and then mixed modes of feeding; FF-2-3 - Formula Food for the first 2 to 3 months followed by formula and/or solid food; FF/SF - Parallel use of formula or solid food since the first month; FAS -Food allergy symptomatic children; DDFA – Doctors’ diagnosed food allergy

In IFPSII, information on various infant feeding patterns were collected on monthly interval basis from months two to seven and then, every 7 weeks until month 12. The information for the respective month was corrected for the age of the infant when the questionnaire was returned [32–34]. Based on latent transition analyses, described in detail elsewhere [32, 35], we classified a changing feeding pattern over time into following categories: *Direct feeding at the breast (****DBF-3m****)*, i.e., feeding directly at the breast for at least 3 months, not including pumping methods or any other additional food or liquid, followed by mixed feeding for the next 3 months; this group constituted our reference group; *Direct feeding at the breast as well as pumping and feeding (****DBF/BM****)* includes direct feeding at the breast and feeding of stored breast milk for the first 3 months, followed by mixed feeding; *Concurrent application of direct feeding at the breast, pumping and feeding and formula feeding* ***(DBF/BM/FF)***, in the first 6 months; *Direct feeding at the breast for a month and then mixed modes of feeding (****DBFShort****); Formula Food (****FF-2-3****)* includes formula for the first two to 3 months followed by formula and/or solid food; and *Formula and Solid Food (****FFSF****)*, described parallel use formula or solid food since the first month (Fig. 1).

### Outcome variables

Information on food allergy symptoms and doctors’ diagnosed food allergy was collected four times at four, nine, 12 months, and at 6 years. We defined two outcome variables: (i) Any food allergy related symptoms (FAS) and (ii) Doctors’ diagnosed food allergy (DDFA). The rationale for using two outcome variables is that only a small proportion of symptomatic children were taken to the doctor for diagnosis in our study.

Presence or absence of any symptoms in the infants constituted the FAS outcome variable. We defined FAS from IFPSII using the question “What symptoms of a problem with food has your baby had?” at four, nine and 12 months. In Y6FU questionnaire, “What symptoms has your 6-year-old had because of a reaction to food?” was used to determine FAS. The list of symptoms is provided in the Additional file 1: Table S1. Eighteen symptoms were listed and more than one symptom could be reported. We also investigated if symptoms in infancy persisted to later time period.

DDFA was determined in IFPSII at months four, nine and 12, using the following two questions: “Did you take your baby to a medical doctor because of these problems with food?” (months 4 and 12) or “Have you taken your baby to a medical doctor because of these problems with food since he or she was 4 months old?” (month 9) and “Was your baby diagnosed by a medical doctor as having an allergy to any food?” In the Y6FU questionnaire, the two questions, “Has your 6-year-old ever been taken to a medical doctor because of a possible food allergy?” and “Has your 6-year-old been diagnosed by a doctor as having an allergy to any food?” had the options, “No”, “Yes, within the past 12 months” and “Yes, more than 12 months ago”. The latter two options were coded as doctoral visit. Then, if a medical diagnosis was given, DDFA was considered to be present.

In addition, at each time point, we investigated if the food allergy symptomatic children were taken to a physician for diagnosis. Among those children with symptoms and those diagnosed with a food allergy we investigated what tests were performed for the diagnosis of food allergy using, “If your baby was tested or examined for food allergy, what method was used? (months four, nine, and 12),” or “What testing method was used by a doctor to check for a food allergy? (Year 6).” Description of symptoms, skin test, blood test, esophageal or intestinal study, food elimination, food challenge, other tests and no test were listed as the options for the diagnostic tests. A mother could select more than one test in the questionnaire.

### Confounders

Maternal race was collected as part of the birth screener, categorized into White, Black, Hispanic, Asia Pacific Islander and Others. Information about maternal education (some grade school, college, graduate, post graduate) was part of the prenatal questionnaire. The prenatal questionnaire also collected data about marital status (married, widowed, divorced, separated, never married/domestic partnership) and maternal smoking during pregnancy. The latter was dichotomized as “Yes” or “No,” based on the question, “On the average, how many cigarettes do you smoke a day now?” Second hand/household smoking was defined in a similar manner: “How many people not including yourself smoke in your house most days? (options: 0, 1, 2, 3 or more),” and maternal and paternal history of food allergy were also collected as part of prenatal questionnaire. Pre-pregnancy maternal body mass index (kg/m^2^) was calculated and classified as normal (18.5 to < 25), overweight (25 to < 30), obese (> 30) and underweight (< 18.5). Information on the birth order of the infants, maternal employment status (full time, part-time, unemployed) were also part of prenatal questionnaire.

The neonatal questionnaire included data about the mode of delivery, characterized as vaginal (induced and not induced) and caesarean (planned and unplanned), baby’s birthweight as well as its sex. The Y6FU questionnaire documented the smoking status of mother when the offspring were 6 years old, and the height (in) and weight (lb) of the 6 year old children. We calculated the BMI (kg/m^2^) of the offspring for months 3, 7, 12 and 72.

### Statistical analysis

All the statistical analyses were performed using SAS/STAT® software, version 9.4 of the SAS system (SAS Institute, Cary, NC, USA). We tested whether the final sample was representative of the initial cohort using the one sample t tests and goodness of fit Chi-squared tests for continuous and categorical variables, respectively. In addition, we also compared the demographic characteristics between those with and without food allergy symptoms and doctors’ diagnosed food allergy using chi-squared/fisher’s exact and two-sample t-tests for continuous and categorical variables, respectively. Since we had repeated measurements over multiple time points, we used linear models with parameters estimated via Generalized Estimating Equations (GEE) to infer relative risks of FAS and DDFA in the first 6 years controlling for potential confounders (sex of the offspring, maternal smoking during pregnancy, mode of delivery, race/ethnicity, maternal education, and maternal and paternal history of food allergy). GEE provides a practical way to analyze discrete correlated data from repeated measurements across different time points [36, 37]. Since FAS and DDFA are rare events, our data was modeled using log link function with Poisson distribution.

## Results

A total of 1387 participants had complete IFP data and information on food allergy symptoms (FAS) and doctors’ diagnosed food allergy (DDFA) at 6 years of age. We did not identify any differences in the demographic characteristics between our analytical sample (*n* = 1387) and the initial cohort (*n* = 1542) (Table 1). Among 1387 mothers who had complete IFP data, 87.1% of mothers were white, 3.7% were black, 5.0% were Hispanic, 6.2% consisted of all other races. Regarding education, 68.6% had some college education but no degree (1–3 years) or were college graduates; 80.0% were married and 68.6% were full time employees; 88.8% reported no smoking in the household. There was no significant difference between our analytical sample (*n* = 1387) and the study sample (*n* = 1542) for the covariates (Table 1). The sample includes mother-infant pairs from 49 states across the United States.

**Table 1:** Comparison of demographic characteristics and covariates between the initial cohort and the analytical cohort

Variable	*N* = 1542 (%)	*N* = 1387 (%)	One-sample test *p* - value
Race / ethnicity			
White	85.0	87.1	0.92
Black	3.6	3.7	
Hispanic	5.5	5.0	
Asia/Pacific Islander	2.4	2.4	
Other	1.6	1.8	
Missing/No answer	1.9	2.0	
Marital status			
Married	79.9	80.0	0.99
Widowed	0.2	0.1	
Divorced	1.9	1.9	
Separated	0.8	0.8	
Never Married	12.7	12.6	
Missing/No answer	4.5	4.6	
Delivery mode			
Vaginal (induced and not induced)	70.1	69.8	0.90
C-Section (planned and unplanned)	29.7	30.1	
Missing/No answer	0.1	0.1	
Baby’s sex			
Boy	49.9	49.6	0.85
Girl	49.9	50.3	
Missing/No answer	0.1	0.1	
Education			
Grade School	0.3	0.3	1.00
High School	15.5	15.3	
College	68.6	68.6	
Post-graduate	11.0	11.0	
Missing/No answer	4.6	4.8	
Food allergy history			
Maternal - Yes	7.3	7.2	0.99
No	78.2	78.4	
Missing/No answer	14.5	14.4	
Paternal - Yes	5.5	5.6	0.98
No	80.0	80.0	
Missing/No answer	14.5	14.4	
Prenatal smoking			
Yes	7.4	7.5	0.99
No	92.3	92.2	
Missing/No answer	0.3	0.3	
Household smoking			
Yes	10.6	11.0	0.76
No	89.2	88.8	
Missing/No answer	0.1	0.1	
Maternal BMI -pre-pregnancy			
Underweight (< 18.5)	4.2	4.3	0.98
Normal (18.5 to < 25)	43.1	42.4	
Overweight (25 to < 30)	25.3	25.5	
Obese (> 30)	24.5	24.9	
Missing/No answer	3.0	2.9	
Maternal employment			
Full time	70.2	69.7	1.00
Part time	12.1	12.1	
Unemployed	6.5	6.7	
Missing/No answer	11.2	11.5	
Birth order			
First born	41.4	41.3	1.00
Second born	18.0	18.0	
Third born or more	38.8	38.9	
Missing/No answer	1.8	1.8	
Smoking at Y6FU			
Yes	11.4	11.1	0.99
No	87.6	87.8	
Missing/No answer	1.0	1.1	
Infant’s birthweight (pounds)	7.7	7.7	0.33
6-year BMI (kg/m^2^)	16.6 ± 3.3	16.6 ± 3.2	0.95

Confounders include maternal and paternal history of allergy, pre-pregnancy smoking, baby’s sex, offspring BMI at infancy and 6-years of age, and delivery mode

Categorical variables were tested using chi-square goodness of fit test

Continuous variables were tested using one-sample t-test

About 10% of children with food allergy symptoms had a paternal history of food allergy. Among those children who were diagnosed by a doctor with food allergy, a higher proportion were black or of Hispanic ethnicity (Additional file 2: Table S2). Food allergy symptoms included stomach cramps, vomiting, diarrhea, constipation, flushing among others; Additional file 1: Table S1 shows this in more detail. Most commonly reported symptoms were gastro-intestinal or skin rash related. However, mothers often visited a doctor when the child had trouble breathing, loss of consciousness, blood in stool, and/or swollen eyes/lips. For example, at month nine, two children were reported to have had trouble breathing, of which, both were taken to the physician and diagnosed as having food allergy, on the other hand, 55 children had a skin rash, of which, 21 were taken to the doctor and nine were diagnosed with food allergy (Additional file 1: Table S1).

In total, 194 infants (14%) were directly breastfed for first 3 months, 253 (18.2%) were fed at the breast and via a bottle, 323 (23.3%) experienced mixed modes of feeding from the beginning, 122 (8.8%) were directly breastfed for 1 month followed by other feeding modes, 364 (26.2%) received formula for 3 months, and 131 (9.4%) formula and solid food within the first 3 months (Table 2). The crude prevalence of FAS and DDFA was highest among children who were exposed to mixed feeding (26.8 and 30.3%, respectively) and formula feeding (26.2 and 28.8%, respectively, Table 2).

**Table 2:** Infant feeding patterns and food allergy symptoms and diagnosis

Infant Feeding Patterns (*n* = 1387)*						
Outcome Variables	DBF-3m *n* = 194, 14% *n* (%)	DBF/BM *n* = 253, 18.2% *n* (%)	DBF/BM/FF *n* = 323, 23.3% *n* (%)	DBFShort *n* = 122, 8.8% *n* (%)	FF-2-3 *n* = 364, 26.2% *n* (%)	FFSF *n* = 131, 9.4% *n* (%)
Food Allergy Symptomatic Children (*n* = 328)	37 (11.3)	64 (19.5)	88 (26.8)	26 (7.9)	86 (26.2)	27 (8.2)
Doctors’ Diagnosed Food Allergy (*n* = 76)	6 (9.1)	8 (12.1)	30 (30.3)	7 (10.6)	19 (28.8)	6 (9.1)

*DBF-3m* Direct feeding at the breast i.e., feeding directly at the breast for at least 3 months, not including pumping methods or any other additional food or liquid, followed by mixed feeding; this group constituted our reference group, *DBF/BM* Direct feeding at the breast as well as pumping and feeding includes direct feeding at the breast and feeding of stored breast milk (BM) for the first 3 months, followed by mixed feeding, *DBF/BM/FF* Concurrent application of direct feeding at the breast, pumping and feeding and formula feeding in the first 6 months, *DBFShort* Direct feeding at the breast for a month and then mixed modes of feeding, *FF-2-3* Formula food for the first 2 to 3 months followed by formula and/or solid food, *FFSF* Parallel use of formula or solid food since the first month

*Analytical cohort

Controlling for confounders, children at four, nine, 12 months and 6 years who were exposed to mixed feeding (DBF/BM/FF) after birth had 1.54 times the risk (95% CI 1.04, 2.29) of FAS compared to the group of children who were fed at the breast for 3 months (Table 3). Although not significant, children who were fed formula in the first two to 3 months and then solid food and formula food (FFSF), tended to be at a higher risk (RR 1.34, 95% CI 0.89, 2.02).

**Table 3:** Risk ratios and their 95% confidence intervals for food allergy symptomatic children and diagnosed children

Variables	Food allergy symptoms (*N* = 326)		Doctors’ diagnosed food allergy(*N* = 76)	
	Risk ratio (95% CI)	*p* value	Risk ratio (95% CI)	*p* value
Direct feeding at the breast for 3 months (DBF-3m)	1.00 (reference)	–	1.00 (reference)	–
Pumping and feeding for the first 3 months (DBF/BM)	1.22 (0.81, 1.83)	0.34	1.01 (0.24, 4.28)	0.99
Mixed modes of feeding from the beginning (DBF/BM/FF)	1.54 (1.04, 2.29)	0.03*	2.13 (0.69, 6.53)	0.19
Direct feeding at the breast for 1 month (DBFShort)	1.05 (0.64, 1.72)	0.84	1.79 (0.45, 7.06)	0.41
Formula feeding for 3 months (FF-2-3)	1.34 (0.89, 2.02)	0.16	2.04 (0.69, 6.07)	0.20
Formula and solid food from the start (FFSF)	1.09 (0.61, 1.95)	0.78	1.89 (0.42, 8.44)	0.41
Maternal Allergy	1.02 (0.71, 1.49)	0.90	2.13 (0.98, 4.64)	0.06
Paternal Allergy	1.36 (1.01, 1.83)	0.04*	2.39 (0.95, 6.04)	0.06
Offspring sex: Boy	0.90 (0.72, 1.12)	0.33	0.95 (0.50, 1.78)	0.86
Prenatal smoking	1.45 (0.97, 2.15)	0.07	2.97 (1.53, 5.79)	0.001*
Caesarian section	0.98 (0.77, 1.24)	0.85	0.97 (0.86, 1.09)	0.62
Off spring BMI (kg/m^2^)	0.98 (0.93, 1.02)	0.30	1.27 (0.68, 2.40)	0.45

*DBF-3 m* Direct feeding at the breast i.e., feeding directly at the breast for at least 3 months, not including pumping methods or any other additional food or liquid, followed by mixed feeding; this group constituted our reference group, *DBF/BM* Direct feeding at the breast as well as pumping and feeding includes direct feeding at the breast and feeding of stored breast milk (BM) for the first 3 months, followed by mixed feeding, *DBF/BM/FF* Concurrent application of direct feeding at the breast, pumping and feeding and formula feeding in the first 6 months, *DBFShort* Direct feeding at the breast for a month and then mixed modes of feeding, *FF-2-3* Formula Food for the first 2 to 3 months followed by formula and/or solid food, *FFSF* Parallel use of formula or solid food since the first month

*significant at alpha = 0.05; 95% CI – 95% confidence interval

Among children diagnosed by a physician with a food allergy, all feeding modes except pumping and feeding showed an increased relative risk although *statistically insignificant*. Children who received mixed (DBF/BM/FF) infant feeding were at 2.13 times risk for DDFA (95% CI 0.69, 6.53). Infants exposed to direct feeding at the breast for a month had 1.79 times the risk; children on formula had 2.04 times the risk and those children who received a combination of formula and solid food were at 1.89 times the risk for food allergy (Table 3).

Paternal history food allergy posed a higher relative risk for FAS but not DDFA. For FAS, the paternal allergy relative risk was 1.36 (95% CI 1.01, 1.83). Maternal history of food allergy did not pose as a significant risk for FAS and DDFA. Prenatal maternal smoker had 2.97 times the risk of having a child with DDFA (95% CI 1.53, 5.97) than mothers who did not smoke prenatally.

**Fig. 2:**
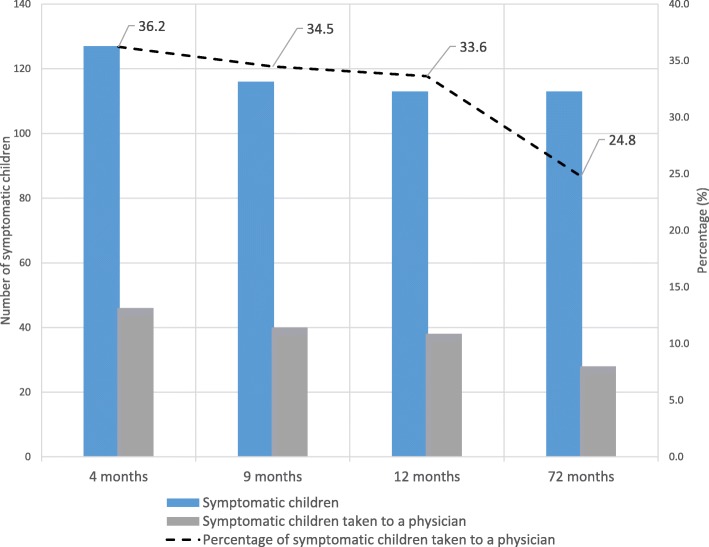
Symptomatic children vs visits to a physician

About 25 to 36% of the symptomatic children were taken to a physician for diagnosis (Fig. 2). Among the 328 children with food allergy symptoms, 52 had persistent symptoms, both at one point in infancy (months four or nine or 12) and at 6 years (Table 4). At 6 years, 51 children had food allergy symptoms for the first time. Fifty-two children with FAS in infancy outgrew their symptoms at 6 years of age. The rest of the symptomatic children (*n* = 173) had sporadic and missing data at one of the four time points hindering further classification. Among the 76 children diagnosed with food allergy, 16 children were diagnosed for the first time at 6 years and 31 children with a prior diagnosis of food allergy no longer had food allergy at 6 years. Data of the remaining children (*n* = 29, symptomatic and diagnosed) had missing information at some time points and thus could not be classified. Four of 16 children who were diagnosed with food allergy for the first time at 6 years had prior symptoms during infancy.

**Table 4:** Classification of incident, persistent and outgrown symptoms and diagnosis

	FAS (*N* = 328)			DDFA (*N* = 76)		
Groups	Infancy (4, 9, 12 months)	Year 6	*n*	Infancy (4, 9, 12 months)	Year 6	*n*
Incident cases at Year 6	–	+	51	–	+	16
Persistent to Year 6	+	+	52	+	+	0
Outgrown food allergy at anytime	+	–	52	+	–	31
Missing/Ambiguous	./+/−	./+/−	173	./+/−	./+/−	29

*FAS* Food Allergy Symptomatic Children, *DDFA* Doctors’ Diagnosed Food Allergy

+ - Presence of Symptoms/Diagnosis; − - Absence of Symptoms/Diagnosis

Mothers reported diagnostic tests performed on their symptomatic children. In infancy (months four, nine and 12) 65 to 68% of food allergy symptomatic children did not have any information on the diagnostic tests. Only 1.8 to 3.2% of children with symptoms underwent a food elimination and food challenge tests in infancy. Additionally, 14.7 to 18% of the mothers reported that their symptomatic infants were not tested for food allergy. At 6 years of age, a majority of symptomatic children underwent a food challenge or food elimination tests with other diagnostic tests, Additional file 3: Table S3 shows this in more detail.

The most common diagnostic method used for diagnosed in infants was food elimination challenge either by itself or in combination with other tests. About 4 to 29.4% of infants, who were reported to have been diagnosed by a physician, did not undergo any diagnostic tests. Among diagnosed six-year olds, the most common diagnostic method used was either a skin prick test or a food elimination challenge. Both of these tests were used alone or in combinations with other tests for diagnosis of food allergy, Additional file 4: Table S4 shows this in more detail.

## Discussion

Food allergy related information was collected at four, nine and 12 months, and at 6 years. We included six types of feeding patterns. The feeding groups are based on empirical developments of infant feeding using latent class transition analyses (LTA) [32, 35]. LTA assesses the transition probabilities between different infant feeding classes (direct feeding at the breast, pumping and feeding, and other methods such as formula or solid food feeding) over time [32]. The patterns identified [32], similar to another study [30], did not reflect the traditional infant feeding patterns such as “exclusive breastfeeding duration,” but reflect the complex settings of infant feeding. There may be other feeding patterns that our study did not cover, however, the purpose of the study was to investigate most common infant feeding patterns and assess the risk of food allergy. To this end, we demonstrated that mixed feeding resulted in increased risk of food allergy symptoms. We did not identify any statistically significant risk for doctors’ diagnosed food allergy with feeding patterns.

Our results show that children had a 57% increased risk for food allergy symptoms at 6 years, when exposed to mixed modes of feeding compared to children who were fed directly at the breast for 3 months, adjusting for confounding. One explanation is that the tolerogenic response of the immune system is food-specific. For example, it was reported that early introduction of wheat, rye, oats, fish, peanuts and eggs decreases the risk of atopic sensitization in childhood but the same was not the case with foods such as milk, sesame seeds and other cereals [13, 15, 38].

Another explanation is that fresh breastmilk is rich in immunoglobulins such as secretory IgA (sIgA), IgG and IgM, and lactoferrin, complementary proteins, macrophages, and T and B lymphocytes to name a few [39]. However, some of the immunological properties present in breastmilk such as IgA and sIgA have been reported to be reduced in stored milk (pumping and feeding), potentially increasing the susceptibility to food antigen sensitization [40]. Specifically, the cellular components such as macrophages and neutrophils were reduced when breast milk was stored for 48 h at 0 to 4 °C. The fat content diminished in hours of letting the breast milk rest at room temperature. Heat treatment additionally reduced sIgA and enzymatic activity of breastmilk [41, 42]. Furthermore, these immune factors are lacking in formula [43].

It is also possible that a mixed fed infant may have been devoid of the mother’s favorable microbiome from direct skin contact that is known to confer protection against atopic sensitization [29]. Additionally, Riskin et al., suggested the possibility of two-way immune transference in that a sick nursing infant may cause an increased production of immune cells such as macrophages and TNF-α in the breastmilk which may be transferred to the infant when nursing, and strengthen the immune system [42]. A mixed fed infant may not have been fed at the breast to experience such benefit. On a different note, Soto-Ramirez et al. suggested the possibility that the introduction of multiple sources of feeding in addition to feeding at the breast may result in atopic sensitization [29]. Given that a seemingly high proportion of mothers practice mixed feeding in our study, this type of feeding merits further investigation.

We did not identify statistically significant feeding risks for DDFA, possibly because the case group consisted of a small number (*n* = 76). Our results, despite a different classification of feeding patterns, agree with the latest report from the American Academy of Pediatrics where the association between the duration of breastfeeding and the incidence of food allergy in early childhood was deemed inconclusive [15]. It was interesting to note that when compared to children who were fed exclusively at the breast for 3 months all other types of feeding patterns with the exception of bottled milk for 3 months showed an increased risk of food allergy. One study suggests that longer duration of exclusive breastfeeding (fed at the breast and bottled human milk) provides a protective effect against atopic sensitization by the age of 5 years [13]. Another prospective birth cohort study suggested that exclusive breastfeeding (fed at the breast and bottled human milk) for a period at least 4 months was related to a lower prevalence of adverse reactions to food compared to children who were exclusively breastfed for less than 4 months. Additionally, partial breastfeeding (breast milk and infant formula/other formulas/solid food) did not reduce the risk of atopic sensitization or allergy related symptoms, which is also in agreement with our findings: among children who were partially breastfed for at least 6 months, 20% showed symptoms of allergies compared to 20% of children who were partially breastfed for more than 6 months [28]. Our results on doctors’ diagnosed food allergy, although statistically insignificant, are in agreement with the above studies.

We also noted that only 25 to 36% of children with food allergy symptoms were taken to a physician for diagnoses. From our analysis, approximately 16% of children had persistent food allergy symptoms during infancy and at 6 years; 16% outgrew their infancy symptoms at 6 years. It is possible that symptoms related to food, though a food allergy, were resolved before further testing [44–46]. Additionally, inadequate awareness and adverse economic restriction of the parents may have hindered them to consult a physician [47]. The gap between food allergy symptoms and report of doctors’ diagnoses of food allergy may be abridged by developing consensual approaches such as standardized questionnaires to investigate the prevalence of food allergies in children (symptoms and medical diagnoses).

About 65 to 68% of food allergy symptomatic children did not have any information on the diagnostic tests. However, this finding is not unusual. For instance, in the Australian HealthNuts study, at 4 years of age, 82.8% of the children with food allergy symptoms did not participate in oral food challenge [48]. Since food allergies is a growing public health concern, it is imperative to consider the results of both, food allergy questionnaires and of specific oral challenge tests with similar importance.

Only a small proportion of children with symptoms of food allergy underwent comprehensive testing in our study. Among the 328 children with FAS only 18 to 27.4% had specific diagnostic tests in infancy. However, 67.7% of symptomatic 6 year olds took diagnostic tests. Over a 26 million adults (10.8%) in the US have had at least one severe food allergic reaction [49], the occurrence of symptoms of food allergy in infancy and childhood, therefore, may provide valuable information on onset of food allergies.

A parental history of food allergy, in particular paternal, was a significant risk factor for the incidence of food allergy symptoms but not in diagnosed children. Maternal history of food allergy did not pose as a risk factor for symptoms nor diagnosed food allergy. We do not have an explanation as to why a paternal history of food allergy increased the risk of food allergy symptoms. However, the finding that maternal history of food allergy is not associated with increased risk of food allergy is not surprising. Several prospective studies have shown that a mother’s dietary exposure is not associated with prevention of atopic sensitization in the offspring [15, 50].

Interestingly, we found that prenatal smoking of the mother was related to a higher risk of doctors’ diagnosed food allergy in the offspring. This association has not been frequently investigated. We only found one study that reported an increased risk of prenatal smoking of the mother resulting in an increased likelihood of sensitization to food allergens (Odds ratio: 2.2, 95% CI 1.1, 4.2) [51].

A strength of this investigation is the longitudinal follow-up in close intervals in the first year and then at 6 years. Our study is also novel since it addresses complex patterns of exposure to different food sources of infant feeding during the first 6 months of life. In addition to including a matrix of feeding exposure changing over time we also differentiated between direct feeding at the breast and pumping and feeding of bottled milk. Another strength of the study is the inclusion of infants who were older than 35 weeks at gestation, singleton, and had a birthweight of over 5 lb. possibly excluding small for gestational age infants, making our results applicable to a specific selected population.

One limitation is that food allergy symptoms in the study are self-reported and could be confused by the parents with mild food poisoning or food intolerance. Nevertheless, it must be acknowledged that the symptoms affect the quality of life of the children and the parents [52, 53]. Hence, there is a need to improve approaches to ascertain the true incidence/prevalence of food allergy symptoms among children [54, 55]. Another limitation is the possible over-reporting of direct breastfeeding leading to a potential misclassification. However, this misclassification is expected to be similar in both the children with and without symptoms, as well as in the diagnosed and the undiagnosed, potentially resulting in a non-differential misclassification. A third limitation is that a majority of our study population are white and had some college education affecting the extrapolation of our results to other groups.

## Conclusion

The analysis of Infant Feeding Practices Study II and its 6-year follow up suggests that mixed mode of feeding (direct feeding at the breast, pumping and feeding, and formula feeding) in the first 3 months poses an increased risk for food allergy symptoms in early childhood. Interestingly, compared to direct feeding at the breast for 3 months, formula feeding does not increase the risk for food allergy in children. We consider that not a single source of infant feeding, but exposure to multiple sources of foods in the first 3 months may lead to increased risk of allergic symptoms. Regarding the assessment of food allergy, future efforts are needed to determine consensual approaches such as standardized questionnaires to investigate the prevalence of food allergies in children (symptoms and medical diagnoses).

## Supplementary information

**Additional file 1:** **Table S1.** Symptoms of food allergy considered in infants and at 6 years of age. List of all the symptoms collected with regards to food allergy at all four time points. The table shows the most commonly reported symptoms.

**Additional file 2:** **Table S2.** Demographic characteristics differences between those with food allergy outcomes and those without. This table provides information on the differences in important demographic covariates between those with and without food allergy symptoms and doctors’ diagnosed food allergy at any time point of the study period.

**Additional file 3:** **Table S3.** Diagnostic tests reported by mothers in children with food allergy symptoms. This table provides information on the different diagnostic tests that symptomatic children underwent during infancy (months 4, 9, 12) and at 6 years of age.

**Additional file 4:** **Table S4.** Diagnostic tests reported by mothers in children diagnosed with food allergy. This table provides information on the different diagnostic tests that diagnosed children underwent during infancy (months 4, 9, 12) and at 6 years of age.

## Data Availability

The datasets generated and/or analyzed during the current study are available in the CDC repository, [https://www.cdc.gov/breastfeeding/data/ifps/index.htm].

## Abbreviations

95 % CI: 95 % Confidence Intervals
BMI: Body Mass Index (kg/m^2^)
CDC: Centers for Disease Control and Prevention
DBF/BM: Pumping and feeding for the first 3 months followed by mixed feeding
DBF/BM/FF: Concurrent application of feeding at the breast, pumping and feeding and formula feeding for the first 6 months (mixed feeding)
DBF-3 M: Direct feeding at the breast for 3 months followed by mixed feeding
DBFShort: Direct feeding at the breast for 1 month followed by mixed feeding
DDFA: Doctors’ Diagnosed Food Allergy
FAS: Food Allergy Symptoms
FDA: United States Food and Drug Administration
FF-2-3: Formula feeding for 3 months followed by mixed, or formula or solid food feeding
FFSF: Formula and solid food from the first month of infant feeding
GEE: Generalized Estimating Equations
IFP: Infant Feeding Patterns
IFPSII: Infant Feeding Practices Study II
LTA: Latent Transition Analyses
RR: Risk Ratio
sIgA: Secretary Immunoglobulin A
Y6FU: Year 6 Follow Up

## References

[1] Lack G (2012). Update on risk factors for food allergy. J Allergy Clin Immunol.

[2] Allen KJ, Koplin JJ (2016). Prospects for prevention of food allergy. J Allergy Clin Immunol Pract.

[3] Yu W, Freeland DMH, Nadeau KC (2016). Food allergy: immune mechanisms, diagnosis and immunotherapy. Nat Rev Immunol.

[4] Branum AM, Lukacs SL (2008). Food allergy among U.S. children: trends in prevalence and hospitalizations. NCHS Data Brief.

[5] Hill DA, Grundmeier RW, Ram G, Spergel JM (2016). The epidemiologic characteristics of healthcare provider-diagnosed eczema, asthma, allergic rhinitis, and food allergy in children: a retrospective cohort study. BMC Pediatr.

[6] US Food and Drug Administration. Nutrition: Center for Food Safety and Applied Nutrition. Food allergies: what you need to know there. Available from: https://www.fda.gov/consumers/consumer-updates/food-allergies-reducing-risks. Accessed 18 Oct 2019.

[7] Nurmatov U, Devereux G, Sheikh A (2011). Nutrients and foods for the primary prevention of asthma and allergy: systematic review and meta-analysis. J Allergy Clin Immunol.

[8] Arshad SH (2005). Primary prevention of asthma and allergy. J Allergy Clin Immunol.

[9] McGowan EC, Keet CA (2014). Primary prevention of food allergy in children and adults: systematic review. Pediatrics.

[10] Sicherer SH, Muoz-Furlong A, Sampson HA (2004). Prevalence of seafood allergy in the United States determined by a random telephone survey. J Allergy Clin Immunol.

[11] Sicherer SH, Sampson HA (2010). Food allergy. J Allergy Clin Immunol.

[12] Sicherer SH, Wood RA, Stablein D, Lindblad R, Burks AW, Liu AH (2010). Maternal consumption of peanut during pregnancy is associated with peanut sensitization in atopic infants. J Allergy Clin Immunol.

[13] Nwaru BI, Takkinen H-M, Niemel O, Kaila M, Erkkola M, Ahonen S (2013). Timing of infant feeding in relation to childhood asthma and allergic diseases. J Allergy Clin Immunol.

[14] Joseph CLM, Ownby DR, Havstad SL, Woodcroft KJ, Wegienka G, MacKechnie H (2011). Early complementary feeding and risk of food sensitization in a birth cohort. J Allergy Clin Immunol.

[15] Greer FR, Sicherer SH, Burks AW (2019). The effects of early nutritional interventions on the development of atopic disease in infants and children: the role of maternal dietary restriction, breastfeeding, hydrolyzed formulas, and timing of introduction of allergenic complementary foods. Pediatrics.

[16] Friedman NJ, Zeiger RS (2005). The role of breast-feeding in the development of allergies and asthma. J Allergy Clin Immunol.

[17] Dieterich C, Felice JP, Sullivan E, Rasmussen KM (2013). Breastfeeding and health outcomes for the mother-infant dyad. Pediatr Clin N Am.

[18] Kramer MS (2011). Breastfeeding and allergy: the evidence. Ann Nutr Metab.

[19] Venter C, Maslin K, Dean T, Arshad SH (2016). Does concurrent breastfeeding alongside the introduction of solid food prevent the development of food allergy?. J Nutr Sci.

[20] Smith HA, Becker GE (2016). Early additional food and fluids for healthy breastfed full-term infants. Cochrane Database Syst Rev.

[21] Kramer MS, Matush L, Vanilovich I, Platt R, Bogdanovich N, Sevkovskaya Z (2007). Effect of prolonged and exclusive breast feeding on risk of allergy and asthma: cluster randomised trial. BMJ.

[22] Abrams EM, Becker AB (2015). Food introduction and allergy prevention in infants. CMAJ.

[23] Goldsmith AJ, Koplin JJ, Lowe AJ, Tang ML, Matheson MC, Robinson M (2016). Formula and breast feeding in infant food allergy: a population-based study. J Paediatr Child Health.

[24] World Health Organization (1991). Indicators for assessing breastfeeding practices.

[25] Labbok MH, Belsey M, Coffin CJ (1997). A call for consistency in defining breast-feeding. Am J Public Health.

[26] Aarts C, Kylberg E, Hornell A, Hofvander Y, Gebre-Medhin M, Greiner T (2000). How exclusive is exclusive breastfeeding? A comparison of data since birth with current status data. Int J Epidemiol.

[27] Heinig MJ, Dewey KG (1996). Health advantages of breast feeding for infants: a critical review. Nutr Res Rev.

[28] Kull I, Wickman M, Lilja G, Nordvall SL, Pershagen G (2002). Breast feeding and allergic diseases in infants-a prospective birth cohort study. Arch Dis Child.

[29] Soto-Ramírez N, Kar S, Zhang H, Karmaus W (2017). Infant feeding patterns and eczema in children in the first 6 years of life. Clin Exp Allergy.

[30] Klopp A, Vehling L, Becker AB, Subbarao P, Mandhane PJ, Turvey SE (2017). Modes of infant feeding and the risk of childhood asthma: a prospective birth cohort study. J Pediatr.

[31] Moreno MA (2016). Atopic diseases in children [patient page]. JAMA Pediatr.

[32] Karmaus W, Soto-Ramírez N, Zhang H (2017). Infant feeding pattern in the first six months of age in USA: a follow-up study. Int Breastfeed J.

[33] Fein SB, Labiner-Wolfe J, Shealy KR, Li R, Chen J, Grummer-Strawn LM (2008). Infant feeding practices study II: study methods. Pediatrics.

[34] Grummer-Strawn LM, Li R, Perrine CG, Scanlon KS, Fein SB (2014). Infant feeding and long term outcomes: results from the year 6 follow-up of children in the infant feeding practices study II. Pediatrics.

[35] Velicer WAF, Artin RAAM, Collins LM (1996). Latent transition analysis for longitudinal data. Addiction.

[36] Greenland S (1989). Modelling and variable selection in epidemiologic analysis. Am J Public Health.

[37] Johnston G, Stokes M (1999). Repeated measures analysis with discrete data using the SAS® system.

[38] Mermiri D-ZT, Lappa T, Papadopoulou AL (2017). Review suggests that the immunoregulatory and anti-inflammatory properties of allergenic foods can provoke oral tolerance if introduced early to infants’ diets. Acta Paediatr.

[39] Orlando S (1995). The immunologic significance of breast milk. J Obstet Gynecol Neonatal Nurs.

[40] Lawrence RA (1999). Storage of human milk and the influence of procedures on immunological components of human milk. Acta Paediatr.

[41] Van Zoeren-Grobben D, Schrijver J, Van den Berg H, Berger HM (1987). Human milk vitamin content after pasteurisation, storage, or tube feeding. Arch Dis Child.

[42] Riskin A, Almog M, Peri R, Halasz K, Srugo I, Kessel A (2012). Changes in immunomodulatory constituents of human milk in response to active infection in the nursing infant. Pediatr Res.

[43] Institute of Medicine (2004). Chapter 3: Comparing infant formulas with human milk. Infant formula: evaluating the safety of new ingredients.

[44] Rona RJ, Keil T, Summers C, Gislason D, Zuidmeer L, Sodergren E (2007). The prevalence of food allergy: a meta-analysis. J Allergy Clin Immunol.

[45] Sánchez J, Sánchez A (2015). Epidemiology of food allergy in Latin America. Allergol Immunopathol (Madr).

[46] Grundy J, Matthews S, Bateman B, Dean T, Arshad SH (2002). Rising prevalence of allergy to peanut in children: data from 2 sequential cohorts. J Allergy Clin Immunol.

[47] Gupta RS, Kim JS, Springston EE, Pongracic JA, Wang X, Holl J (2009). Development of the Chicago food allergy research surveys: assessing knowledge, attitudes, and beliefs of parents, physicians, and the general public. BMC Health Serv Res.

[48] Koplin JJ, Allen KJ, Gurrin LC, Peters RL, Lowe AJ, Tang MLK (2013). The impact of family history of allergy on risk of food allergy: a population-based study of infants. Int J Environ Res Public Health.

[49] Gupta RS, Warren CM, Smith BM, Jiang J, Blumenstock JA, Davis MM (2019). Prevalence and severity of food allergies among US adults. JAMA Netw Open.

[50] Greer FR, Sicherer SH, Burks AW (2008). The effects of early nutritional interventions on the development of atopic disease in infants and children: the role of maternal dietary restriction, breastfeeding, hydrolyzed formulas, and timing of introduction of allergenic complementary foods. Pediatrics.

[51] Kulig M, Luck W, Wahn U (1999). The association between pre- and postnatal tobacco smoke exposure and allergic sensitization during early childhood. Hum Exp Toxicol.

[52] Warren CM, Otto AK, Walkner MM, Gupta RS (2016). Quality of life among food allergic patients and their caregivers. Curr Allergy Asthma Rep.

[53] Gupta R, Holdford D, Bilaver L, Dyer A, Holl JL, Meltzer D (2013). The economic impact of childhood food allergy in the United States. JAMA Pediatr.

[54] Peters RL, Koplin JJ, Gurrin LC, Dharmage SC, Wake M, Ponsonby AL (2017). The prevalence of food allergy and other allergic diseases in early childhood in a population based study: HealthNuts age 4-year follow-up. J Allergy Clin Immunol.

[55] Boyce JA, Assaad A, Burks W, Jones S, Sampson H, Wood R (2010). Guidelines for the diagnosis and management of food allergy in the US: Report of the NIAID- sponsored expert panel. J Allergy Clin Immunol.

