# Supplementary material for: The association of infant feeding patterns with food allergy symptoms and food allergy in early childhood
Source: Int Breastfeed J. 2019 Oct 24;14:43. doi: 10.1186/s13006-019-0241-x (PMC6813109; doi:10.1186/s13006-019-0241-x)
Supplement: Supplementary file 1 — Additional file 1: Table S1. Symptoms of food allergy considered in infants and at 6 years of age. List of all the symptoms collected with regards to food allergy at all four time points. The table shows the most commonly reported symptoms. [file 13006_2019_241_MOESM1_ESM.docx]

**Table S1. Symptoms of food allergy considered in infants and at 6 years of age**

| Sx (Yes) | Month 4   (n=1542) | | | Month 9  (n=1542) | | | | Month 12  (n=1542) | | | | Month 72  (n=1542) | | | |
| --- | --- | --- | --- | --- | --- | --- | --- | --- | --- | --- | --- | --- | --- | --- | --- |
|  | Sx present* % (n) | Taken to Doctor % | Dx % | Sx * % (n) | Taken to Doctor % | | Dx % | Sx * % (n) | Taken to Doctor % | | Dx % | Sx * %(n) | Taken to Doctor^# %^ | | Dx % |
| Congestion | 0.90 (14) | 71 | 70 | 0.50 (8) | 37.5 | 100 | | 0.64 (10) | 80 | 75 | | 1.36 (21) | 100 | 80 | |
| Runny nose | 0.40 (6) | 50 | 33 | 0.32 (5) | 80 | 75 | | 0.84 (13) | 77 | 80 | | 1.49 (23) | 100 | 83 | |
| Asthma/Wheeze | 0.20 (3) | 67 | 0 | 0.19 (3) | 100 | 67 | | 0.26 (4) | 75 | 100 | | 0.97 (15) | 100 | 100 | |
| Trouble breathing | 0.06 (1) | 100 | 100 | 0.13 (2) | 100 | 100 | | 0.20 (3) | 100 | 67 | | 1.36 (21) | 100 | 100 | |
| Coughing | 0.40 (6) | 100 | 83 | 0.26 (4) | 75 | 100 | | 0.58 (9) | 88 | 75 | | 1.29 (20) | 100 | 90 | |
| Swollen eyes/lips | 0.30 (4) | 50 | 50 | 0.30 (5) | 20 | 100 | | 0.91 (14) | 64 | 88 | | 1.75 (27) | 100 | 96 | |
| Hives/Welts | 0.30 (5) | 60 | 33 | 1.67 (26) | 26.9 | 42.8 | | 1.75 (27) | 40 | 91 | | 3.20 (50) | 100 | 88 | |
| Flushing | 0.10 (2) | 50 | 100 | 0.26 (4) | 50 | 50 | | 0.65 (10) | 60 | 100 | | 0.80 (12) | 100 | 92 | |
| Skin rash | 1.60 (25) | 60 | 60 | 3.57 (55) | 38 | 43 | | 3.83 (59) | 39 | 69 | | 3.30 (51) | 100 | 86 | |
| Gassiness | 6.30 (98) | 36 | 40 | 3.17 (49) | 35 | 47 | | 2.53 (39) | 38 | 53 | | 2.50 (39) | 100 | 77 | |
| Vomiting | 2.00 (31) | 55 | 42 | 1.43 (22) | 45 | 50 | | 1.75 (27) | 44 | 67 | | 2.14 (33) | 100 | 69.7 | |
| Diarrhea | 1.20 (27) | 63 | 47 | 1.56 (24) | 33 | 63 | | 2.33 (36) | 27 | 60 | | 1.95 (30) | 100 | 80 | |
| Constipation | 1.80 (27) | 52 | 57 | 0.84 (13) | 46 | 50 | | 0.32 (5) | 20 | 100 | | 0.65 (10) | 100 | 80 | |
| Colic | 2.00 (31) | 45 | 29 | 0.40 (6) | 50 | 67 | | 0.40 (6) | 75 | 75 | |  |  |  | |
| Irritability | 5.45 (84) | 42 | 40 | 2.66 (41) | 37 | 53 | | 2.27 (35) | 34 | 58 | | 1.29 (20) | 100 | 95 | |
| Sleeplessness | 1.36 (21) | 52 | 46 | 0.90 (15) | 53 | 50 | | 1.17 (18) | 50 | 44 | | 0.30 (5) | 100 | 100 | |
| Blood in stool | 0.60 (9) | 89 | 75 | 0.20 (3) | 100 | 100 | | 0.10 (2) | 50 | 100 | | 0.30 (5) | 100 | 80 | |
| Loss of consciousness | 0.06 (1) | 100 | 100 | 0.00 (0) | 0 | 0 | | 0.00 (0) | 0 | 0 | | 0.06 (1) | 100 | 100 | |
| Any Symptom | 25 (395) | 49 (195) | 48 (94) | 18.36 (285) | 40.7 (116) | 56.03 (65) | | 20.53 (317) | 45.7 (145) | 71 (103) | | 24.7 (383) | | 1. (329) | |

*- Assuming no other children had the listed symptoms; Sx – Symptoms; Dx - Diagnosed

# - In the year 6 follow up, the reported visit to the doctor included anytime within the past 12 months and greater than the past 12 months
