# Supplementary material for: The association of infant feeding patterns with food allergy symptoms and food allergy in early childhood
Source: Int Breastfeed J. 2019 Oct 24;14:43. doi: 10.1186/s13006-019-0241-x (PMC6813109; doi:10.1186/s13006-019-0241-x)
Supplement: Supplementary file 2 — Additional file 2: Table S2. Demographic characteristics differences between those with food allergy outcomes and those without. This table provides information on the differences in important demographic covariates between those with and without food allergy symptoms and doctors’ diagnosed food allergy at any time point of the study period. [file 13006_2019_241_MOESM2_ESM.docx]

**Table S2.** **Demographic characteristics differences between those with food allergy outcomes and those without**

| **Variable** | **FAS  (N=328)** | **No FAS  (N=1061)** | **Chi-squared tests** | **DDFA (N=76)** | **No DDFA (N=1311)** | **Chi-squared tests** |
| --- | --- | --- | --- | --- | --- | --- |
|  | **(%)** | **(%)** | **p-value** | **(%)** | **(%)** | **p-value** |
| **Race / Ethnicity** | |  |  |  |  |  |
| White | 88.7 | 88.6 | 0.70 | 75.4 | 89.0 | 0.01* |
| Black | 3.8 | 2.6 |  | 9.2 | 2.9 |  |
| Hispanic | 4.1 | 4.5 |  | 7.7 | 4.3 |  |
| Other | 3.5 | 4.3 |  | 7.7 | 3.8 |  |
| **Marital Status** | |  |  |  |  |  |
| Married | 84.4 | 86.6 | 0.58 | 79.0 | 85.3 | 0.21 |
| Widowed | 0.0 | 0.3 |  | 0.0 | 0.2 |  |
| Divorced | 1.9 | 2.0 |  | 0.0 | 2.0 |  |
| Separated | 0.6 | 1.1 |  | 0.0 | 1.0 |  |
| Never Married | 13.1 | 10.0 |  | 21.0 | 11.4 |  |
| **Delivery Mode** | |  |  |  |  |  |
| Vaginal | 67.7 | 68.4 | 0.82 | 62.1 | 68.3 | 0.34 |
| (induced and not induced) |  |  |  |  |  |  |
| C-Section | 32.3 | 31.6 |  | 37.9 | 31.7 |  |
| (planned and unplanned) |  |  |  |  |  |  |
| **Baby's sex** | |  |  |  |  |  |
| Boy | 51.1 | 50.2 | 0.79 | 54.6 | 50.3 | 0.50 |
| Girl | 48.9 | 49.9 |  | 45.4 | 49.7 |  |
| **Education** | |  |  |  |  |  |
| Grade School | 0.0 | 0.2 | 0.75 | 0.0 | 0.2 | 0.89 |
| High School | 13.5 | 15.6 |  | 12.9 | 15.3 |  |
| College | 72.4 | 71.3 |  | 72.6 | 71.4 |  |
| Post-graduate | 14.1 | 13.0 |  | 14.5 | 13.1 |  |
| **Food Allergy History** | | |  |  |  |  |
| Maternal - Yes | 10.4 | 8.8 | 0.46 | 16.1 | 8.1 | 0.05 |
| No | 89.6 | 91.2 |  | 83.9 | 91.9 |  |
| Paternal - Yes | 9.7 | 5.1 | 0.001* | 10.7 | 6.0 | 0.16 |
| No | 90.3 | 95.0 |  | 89.3 | 94.0 |  |
| **Prenatal Smoking** | |  |  |  |  |  |
| Yes | 9.8 | 5.5 | 0.01 | 9.1 | 6.1 | 0.33 |
| No | 90.2 | 94.5 |  | 90.0 | 93.9 |  |
| **6-year off spring BMI (kg/m^2^)** | 16.7±2.8 | 16.6 ±3.3 | 0.41 | 16.8 + 2.6 | 16.5 + 3.1 | 0.50 |

Categorical variables were tested using chi-square goodness of fit test or fisher’s exact

Continuous variables were tested using two-sample t-tests

* - significant at alpha=0.05

FAS – Food allergy symptomatic

DDFA – Doctors’ diagnosed food allergy
