# Supplementary material for: The association of infant feeding patterns with food allergy symptoms and food allergy in early childhood
Source: Int Breastfeed J. 2019 Oct 24;14:43. doi: 10.1186/s13006-019-0241-x (PMC6813109; doi:10.1186/s13006-019-0241-x)
Supplement: Supplementary file 4 — Additional file 4: Table S4. Diagnostic tests reported by mothers in children diagnosed with food allergy. This table provides information on the different diagnostic tests that diagnosed children underwent during infancy (months 4, 9, 12) and at 6 years of age. [file 13006_2019_241_MOESM4_ESM.docx]

**Table S4. Diagnostic tests reported by mothers in children diagnosed with food allergy**

| DDFA | Diagnostic Tests reported by mothers | | | | | | | | Frequency | Percent |
| --- | --- | --- | --- | --- | --- | --- | --- | --- | --- | --- |
|  | Symptom Description | Skin Test | Blood Test | Esophageal Testing | Food Elimination | Food Challenge | Other Tests | Not Examined/Tested |  |  |
| Month 4 | 1 | 1 | 0 | 0 | 0 | 0 | 0 | 0 | 1 | 5.88 |
|  | 1 | 0 | 1 | 0 | 0 | 0 | 0 | 0 | 1 | 5.88 |
|  | 1 | 0 | 0 | 1 | 0 | 0 | 1 | 0 | 1 | 5.88 |
|  | 1 | 0 | 0 | 0 | 1 | 1 | 0 | 0 | 2 | 11.76 |
|  | 1 | 0 | 0 | 0 | 1 | 0 | 0 | 0 | 2 | 11.76 |
|  | 1 | 0 | 0 | 0 | 0 | 0 | 1 | 0 | 3 | 17.65 |
|  | 1 | 0 | 0 | 0 | 0 | 0 | 0 | 0 | 1 | 5.88 |
|  | 0 | 0 | 0 | 0 | 0 | 0 | 0 | 1 | 5 | 29.41 |
|  | 0 | 0 | 0 | 0 | 0 | 0 | 0 | 0 | 1 | 5.88 |
| Month 9 | 1 | 1 | 0 | 0 | 0 | 0 | 0 | 0 | 1 | 5.56 |
|  | 1 | 0 | 1 | 0 | 1 | 1 | 0 | 0 | 1 | 5.56 |
|  | 1 | 0 | 1 | 0 | 1 | 0 | 0 | 0 | 1 | 5.56 |
|  | 1 | 0 | 0 | 0 | 1 | 1 | 0 | 0 | 1 | 5.56 |
|  | 1 | 0 | 0 | 0 | 1 | 0 | 1 | 0 | 1 | 5.56 |
|  | 1 | 0 | 0 | 0 | 1 | 0 | 0 | 0 | 3 | 16.67 |
|  | 1 | 0 | 0 | 0 | 0 | 1 | 0 | 0 | 1 | 5.56 |
|  | 1 | 0 | 0 | 0 | 0 | 0 | 0 | 0 | 3 | 16.67 |
|  | 0 | 1 | 0 | 0 | 0 | 0 | 0 | 0 | 1 | 5.56 |
|  | 0 | 0 | 0 | 0 | 1 | 0 | 0 | 0 | 2 | 11.11 |
|  | 0 | 0 | 0 | 0 | 0 | 0 | 0 | 1 | 3 | 16.67 |
| Month 12 | 1 | 1 | 1 | 0 | 1 | 0 | 0 | 0 | 1 | 4.17 |
|  | 1 | 1 | 0 | 0 | 0 | 0 | 0 | 0 | 3 | 12.50 |
|  | 1 | 0 | 1 | 0 | 1 | 0 | 0 | 0 | 1 | 4.17 |
|  | 1 | 0 | 0 | 0 | 1 | 1 | 0 | 0 | 2 | 8.33 |
|  | 1 | 0 | 0 | 0 | 1 | 0 | 0 | 0 | 2 | 8.33 |
|  | 1 | 0 | 0 | 0 | 0 | 1 | 0 | 0 | 1 | 4.17 |
|  | 1 | 0 | 0 | 0 | 0 | 0 | 1 | 0 | 1 | 4.17 |
|  | 1 | 0 | 0 | 0 | 0 | 0 | 0 | 0 | 4 | 16.67 |
|  | 0 | 1 | 0 | 0 | 0 | 0 | 0 | 0 | 1 | 4.17 |
|  | 0 | 0 | 1 | 0 | 1 | 0 | 0 | 0 | 2 | 8.33 |
|  | 0 | 0 | 0 | 0 | 1 | 0 | 0 | 0 | 3 | 12.50 |
|  | 0 | 0 | 0 | 0 | 0 | 0 | 1 | 0 | 1 | 4.17 |
|  | 0 | 0 | 0 | 0 | 0 | 0 | 0 | 1 | 1 | 4.17 |
|  | 0 | 0 | 0 | 0 | 0 | 0 | 0 | 0 | 1 | 4.17 |
| Year 6 | 1 | 1 | 1 | 0 | 1 | 1 | 0 | 0 | 1 | 4.76 |
|  | 1 | 0 | 1 | 0 | 1 | 0 | 0 | 0 | 1 | 4.76 |
|  | 0 | 1 | 1 | 0 | 1 | 0 | 0 | 0 | 4 | 19.05 |
|  | 0 | 1 | 1 | 0 | 0 | 1 | 0 | 0 | 1 | 4.76 |
|  | 0 | 1 | 1 | 0 | 0 | 0 | 0 | 0 | 3 | 14.29 |
|  | 0 | 1 | 0 | 0 | 1 | 1 | 0 | 0 | 1 | 4.76 |
|  | 0 | 1 | 0 | 0 | 1 | 0 | 0 | 0 | 2 | 9.52 |
|  | 0 | 1 | 0 | 0 | 0 | 0 | 0 | 0 | 4 | 19.05 |
|  | 0 | 0 | 1 | 0 | 1 | 0 | 0 | 0 | 1 | 4.76 |
|  | 0 | 0 | 1 | 0 | 0 | 0 | 0 | 0 | 2 | 9.52 |
|  | 0 | 0 | 0 | 0 | 1 | 1 | 0 | 0 | 1 | 4.76 |

1-Test performed; 0 – No test was performed; DDFA: Doctors’ diagnosed food allergy
